# Albedo feedbacks to future climate via climate change impacts on dryland biocrusts

**DOI:** 10.1038/srep44188

**Published:** 2017-03-10

**Authors:** William A. Rutherford, Thomas H. Painter, Scott Ferrenberg, Jayne Belnap, Gregory S. Okin, Cody Flagg, Sasha C. Reed

**Affiliations:** 1 US Geological Survey, Southwest Biological Science Center, Moab, UT 84532, USA; 2 Joint Institute for Regional Earth System Science and Engineering, University of California, Los Angeles, CA 90095, USA; 3 Department of Geography, University of California, Los Angeles, CA 90095, USA; 4 National Ecological Observatory Network (NEON), Boulder, Colorado 80301, USA

## Abstract

Drylands represent the planet’s largest terrestrial biome and evidence suggests these landscapes have large potential for creating feedbacks to future climate. Recent studies also indicate that dryland ecosystems are responding markedly to climate change. Biological soil crusts (biocrusts) ‒ soil surface communities of lichens, mosses, and/or cyanobacteria ‒ comprise up to 70% of dryland cover and help govern fundamental ecosystem functions, including soil stabilization and carbon uptake. Drylands are expected to experience significant changes in temperature and precipitation regimes, and such alterations may impact biocrust communities by promoting rapid mortality of foundational species. In turn, biocrust community shifts affect land surface cover and roughness—changes that can dramatically alter albedo. We tested this hypothesis in a full-factorial warming (+4 °C above ambient) and altered precipitation (increased frequency of 1.2 mm monsoon-type watering events) experiment on the Colorado Plateau, USA. We quantified changes in shortwave albedo via multi-angle, solar-reflectance measurements. Warming and watering treatments each led to large increases in albedo (>30%). This increase was driven by biophysical factors related to treatment effects on cyanobacteria cover and soil surface roughness following treatment-induced moss and lichen mortality. A rise in dryland surface albedo may represent a previously unidentified feedback to future climate.

Land surfaces in drylands are characterized by sparse, heterogeneous vegetation cover with large interspaces between the vascular plants^1^. In undisturbed settings, these interspaces are predominately colonized by biocrusts, which consist of a diverse soil surface community of cyanobacteria, mosses, lichens, and heterotrophs held together by cyanobacteria filaments^2^,^3^. In many drylands, biocrusts are likely the dominant source of new nitrogen (N) via N_2_ fixation^4^, are a substantial pathway of gaseous N loss^5^, and represent a significant input of carbon (C) via biocrust photosynthesis^6^,^7^.

Recent studies utilizing climate manipulation treatments suggest that climate change may have dramatic effects on biocrust community composition by eliminating key species of mosses and lichens, which are large contributors to biogeochemical and hydrological functions in drylands^8^,^9^,^10^. This climate-induced loss of mosses and lichens in favor of early successional cyanobacteria-dominated biocrusts also reduces the characteristically dark, textured soil surface, which in turn increases the abundance of lighter, smoother surface cover (Fig. 1a–d). Thus, a shift in biocrust community states could cause rapid alteration of dryland albedo and energy balance by returning energy to the atmosphere that was once absorbed by the dark biocrust surfaces^11^,^12^,^13^.

This effect is analogous (although opposite in sign) to climate-induced woody encroachment in high latitude ecosystems, which dramatically alters albedo via the transformation of white, snow-covered landscapes to dark vegetative cover^14^. Despite a growing concern over how climate-driven disturbances will affect biogeophysical processes, little empirical evidence exists regarding how and at what magnitude climate change effects in drylands will create feedbacks to future climate via changes to energy balance.

To evaluate the impact of climate change on dryland energy balance, we assessed short-wave (solar spectrum) albedo in relation to biocrust community composition in 20, 5 m^2^ plots subjected to warming and altered precipitation patterns (supplemental watering). Treatments were applied over 10 years (2005–2014) in a full-factorial design (control, watering, warming, and watering + warming). The warming (+2 °C above ambient for the first three years, then +4 °C for the remaining years) and watering (increased frequency of 1.2 mm summer rainfall events) treatments were selected to meet climate model projections^15^.

A rapid mortality of the dominant moss (*Syntrichia caninervis*), which constituted ~25% of the biocrust community cover in our plots, occurred over the first year of treatments in the watering and watering + warming treatments^8^, while significant moss decline in the warming-only treatment took more than 6 years to emerge^9^. Thus, dramatic mortality of this common moss, which is one of the most abundant desert mosses in the world^16^, was observed with both increased temperature and altered precipitation treatments. Significant responses due to warming and watering + warming were also seen in reduced cover of the dominant lichens (*Collema tenax* and *Collema coccophorum*)^9^. Over time, the decline in moss and lichen species in all treatments produced a shift to a heavily cyanobacteria-dominated biocrust community (i.e., a shift to an early successional state in this system; Fig. 1).

We assessed the effect that this change in community had on albedo by integrating hyperspectral solar-reflectance measurements over four view azimuth and five zenith angles (see Supplementary Information Fig. S5) to account for reflected light scattering in all treatments during the autumn of 2014. Biocrust community composition was determined from point-intercept frames and was used to correlate plot-level albedo with biocrust community cover. We used soil surface roughness and soil moisture [determined via the chain method (see methods) and volumetric water content probes at 2 and 5 cm soil depths, respectively] measured at the time of albedo measurements to investigate the relationships between albedo and climate treatment effects on surface micro-topography, moisture content, and biocrust community structure. Finally, albedo measurements across treatments and communities were combined with global irradiance and biocrust composition distribution data to provide a conceptual estimate of dryland energy balance effects. This novel approach allowed us to join high-resolution albedo measurements with careful assessments of biocrust community composition, micro-topography, and moisture in order to explore, for the first time, how climate-induced changes to biocrust community could affect dryland energy balance.

## Results and Discussion

**Figure 1:**
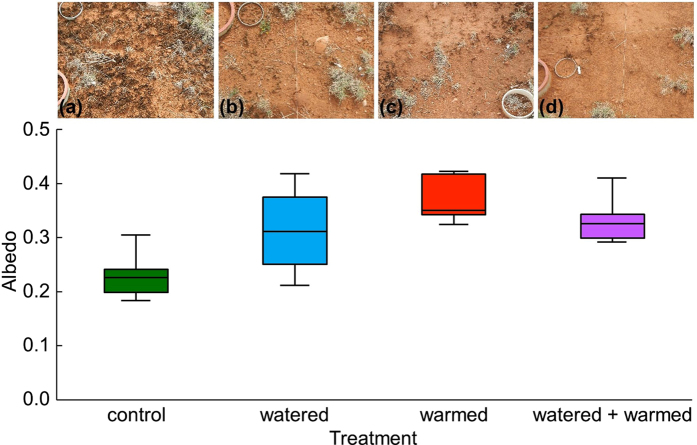
Biocrust cover and albedo by treatment. The photographs (**a**–**d**) illustrate representative effects of the treatments (control, watering, warming, watering + warming) on biocrust cover identified along the horizontal axis corresponding to box and whisker plots. Photographs were taken in areas of little to no vegetation or litter. In the box and whisker panel, boxes show medians for albedo (heavy central line) and 1st and 3rd quartiles; whiskers indicate 1.5 inter-quartile range. Climate manipulation treatments caused a significant increase in albedo of the warming (*P* < 0.01) and warming + watering (*P* < 0.05) treatments compared to untreated controls, but the watering only plots had more variable responses (*P* = 0.06).

Climate change manipulations increased surface albedo by 33% on average across warming, watering, and warming + watering treatments (Fig. 1): a substantial effect measured in a relatively short amount of time (10 years). This change in albedo occurred in large part because climate change treatments drove the mortality of darkly pigmented late succession biocrust populations (i.e., mosses and lichens), which were supplanted by lightly pigmented, cyanobacteria expanding their cover as the biocrust community reverted to an early-successional state^9^.

**Figure 2:**
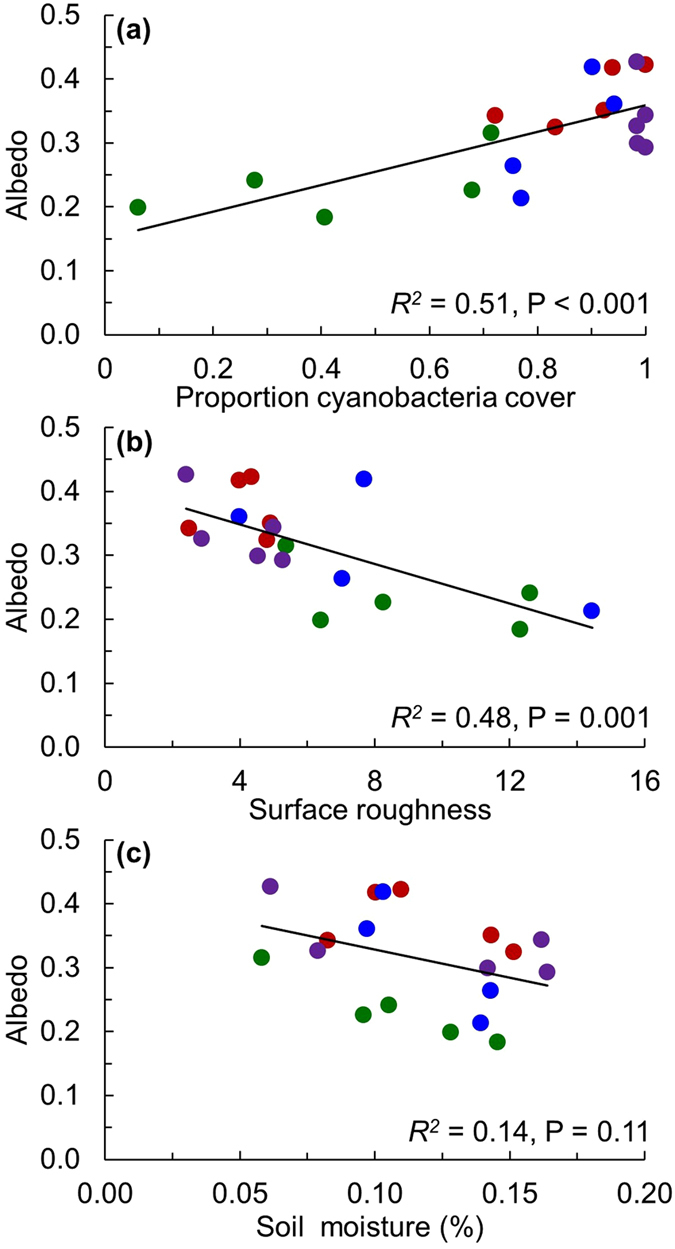
Linear models relating albedo to the proportional cover of cyanobacteria, soil surface roughness and soil moisture. The (**a**) proportional cover of cyanobacteria within biocrust communities of experimental plots was collected in point-intercept frames in autumn 2014, and calculated as the ratio of points intercepting cyanobacteria relative to total biotic cover (sum total of cyanobacteria, moss, and lichen points). Soil surface roughness (**b**) was measured in spring 2014 to calculate a roughness index to characterize the soil surface roughness upslope and across slope within each plot. Soil moisture (**c**) at a depth of 5 cm was measured as the hourly average of values recorded every five minutes during the same time as the albedo measurements. *R*^2^ and *P*-values are from simple linear regression. Climate treatments are denoted by symbol colors (green = control, red = warming, blue = watering, purple = warming + watering). Data from one watered plot was considered an outlier as described in the methods section and was removed from the models shown here (see Supplementary Information Fig. S2 for analyses including the outlier).

A best-fit linear multiple regression model retained cyanobacterial cover, surface roughness, and soil moisture at a 5 cm depth as factors that collectively explained a majority of the variation in plot albedo (*R*^*2*^ = 0.71, P < 0.001). Individual linear regression models suggest that the increase in cyanobacteria cover had the largest effect on albedo (*R*^2^ = 0.51, *P* < 0.001), followed by surface roughness (*R*^2^ = 0.48, *P* = 0.001; Figs 2a and S6), with soil moisture appearing to explain a much smaller (*R*^*2*^ = 0.14) and insignificant (*P* = 0.11) amount of variation in albedo.

Because the absorptive and reflective properties of early and late successional communities are inherently different, it is logical that the community shift observed with warming and altered precipitation treatments greatly altered the soil spectral signatures, and that this change had a large effect on the albedo of this dryland surface. Indeed, with proportional cover of lightly pigmented cyanobacteria explaining over 50% of the variation in albedo across all plots (Fig. 2a), these data suggest a robust control by biocrust community composition in driving dryland soil surface energy balance.

Importantly, biocrusts in some dryland ecosystems are naturally dominated by cyanobacteria, even in late-successional community states (i.e., not dominated by moss and lichens), and the magnitude of biocrust community change effects on albedo would in part depend upon specifics of a site’s community structure. Nevertheless, a recent study of cyanobacteria-dominated biocrusts indicates that as these communities develop, late-successional cyanobacteria species darken the soil surface due to pigmentation linked to the production of UV-absorbing sunscreen-like pigments. These darkly pigmented cyanobacteria species can significantly alter soil temperature (likely via reduced albedo) and affect the overall microbial community^17^. Thus, similar to the degradation of the late successional moss and lichen biocrusts common to our study system, the degradation of dark-colored, late-successional cyanobacteria-dominated biocrusts could also lead to climate change effects on albedo as disturbances favor light-colored, early-successional cyanobacteria species^9^.

**Figure 3:**
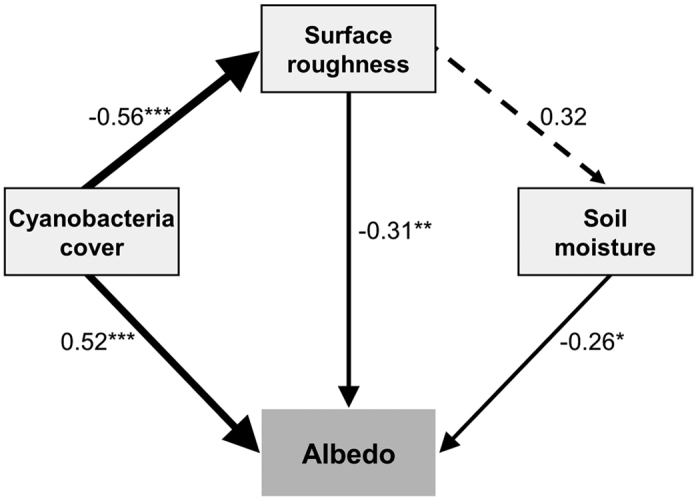
Structural equation model of biotic and abiotic effects on dryland surface albedo. The model (*R*^*2*^ = 0.71) includes the direct effects of proportional cyanobacteria cover, soil moisture, and surface roughness on albedo with the indirect effects of cyanobacteria cover on surface roughness and surface roughness on soil moisture. The arrows represent the unidirectional causal relationships and are scaled to match their standardized effect size. The standardized path coefficients (r) are presented above each arrow. The dashed arrow indicates a clearly, non-significant interaction (P > 0.1) with other arrows P values indicated as *P < 0.1, **P < 0.05, and ***P < 0.01. Data from one watered plot was considered an outlier as described in the methods section and was removed from the model.

While increased cyanobacteria cover with the loss of higher energy absorbers, as in lichen and moss species, was the primary driver of increased albedo under climate treatments in our study system, climate treatments and their interactions with biocrust community composition also altered the soil surface micro-topography and soil hydrology (Figs 1a–d and 2a–c). A best-fit multiple regression model indicated that cyanobacterial cover, surface roughness, and soil moisture collectively explained a substantial amount of the variation in albedo, though cyanobacterial cover and surface roughness were collinear measures (*R*^*2*^ = 0.30, *P* = 0.01). This interaction suggests the presence of potentially complicated mixtures of direct and interacting pathways whereby all three measures (i.e., community, roughness, and moisture) can directly influence albedo (Fig. 3), and where cyanobacterial cover impacts surface roughness. In turn, this change may alter soil moisture through surface to volume effects on water infiltration and evaporation, and together these concomitant changes may collectively help control dryland albedo. However, the way warming treatments affected soil roughness also suggests climate can directly affect albedo of biocrust surfaces via changes to micro-topography, likely via changes to freeze thaw cycles, specifically in cold desert regions where freeze-thaw cycles develop rougher soil surfaces with biocrusts^18^. In particular, the warming treatments significantly reduced the roughness of biocrust soils, while watering alone had a subtler effect (Fig. 2b, also see Supplementary Information Table S1).

To explore this hypothesized causal framework, we constructed a structural equation model (SEM) linking these variables as shown in Fig. 3 19. The SEM explained 71% of the variation in albedo (Full SEM *R*^2^ = 0.71) and fit the observed data well (χ^2^ = 0.63, d.f. = 1, and *P* = 0.43; a smaller χ^2^ and larger *P*-value indicate better SEM fit). The SEM also revealed that changes in albedo with climate treatments were primarily explained by a strong effect of community composition (i.e., as cyanobacterial cover increases, so does albedo), followed by negative direct effects of surface roughness and soil moisture (i.e., as roughness and moisture increase, albedo decreases) (Fig. 3). Notably, cyanobacteria cover also had a strong effect on surface roughness, whereby increases in cyanobacteria cover with the loss of other biocrust species led to a reduction in surface roughness (see also Fig. 2b). As discussed above, warming treatments also directly affected surface roughness (Fig. 2b, Supplementary Information Table S1). The effect of surface roughness on soil moisture was also included in the SEM (Fig. 3, dotted line) to demonstrate that surface roughness does relate to changes in hydrology, but this relationship was not significant.

The simulated warming greatly accelerated soil surface drying, in turn, decreasing the dark, late-successional biocrust species’ cover^8^ and reducing soil surface micro-topography. While the warming prominently affects the soil surface roughness, watering tends to have stronger effects on the total biocrust community composition as seen by Reed *et al*.^8^ (see also Fig. 1b). In all, the climate manipulation treatments induced lighter and smoother soil surfaces and had a direct relationship with the increase in soil surface albedo as seen in Fig. 1. Thus, the treatments showed the potential for a significant dryland negative feedback to future climate via changes to albedo, which resulted from compound disturbances to soil surface biotic and abiotic conditions. The changes to soil surface communities and structure are also likely to affect water infiltration and erosion in ecosystems where productivity and nutrient availability are strongly linked to soil hydrology and stability^2^,^20^. Such feedbacks are currently absent from conceptual and numerical process modeling of future climate, and the present results suggest their inclusion could dramatically improve the scaling, quantifying, and forecasting of climate change.

Community composition shifts that affect albedo in other ecosystems, such as exotic species invasions^21^ or grassland-to-woodland conversions^22^, are often ecosystem dependent, leading to difficulties in scaling these changes from local to regional/global levels^23^. In contrast, the data that exist on biocrust community states suggest that their responses to various disturbances may be remarkably similar when viewed across large latitudinal and longitudinal gradients, even in cases where species identity and composition vary^3^. Thus, the shift in biocrust community composition in response to climate change and the associated impacts on surface albedo of our study system may be strongly indicative of larger transitions in ecosystems with similar biocrust structure, with the magnitude of the effect depending in part on the ecosystem’s biocrust community. While more work is clearly warranted to elucidate this large potential effect, this study demonstrates that dryland surface albedo is highly dependent on biocrust community composition, where the loss of a few key species dramatically alters the energy balance.

Because these data highlight the potential for important climate feedbacks in drylands, we conducted a mathematical exercise to explore the possible scaling implications of our findings. Arid and semiarid ecosystems, collectively termed “drylands”, cover >40% of the Earth’s land surface^24^,^25^ and play a critical role in determining the total global energy budget. Beyond their spatial extent, dryland energy balance dynamics are particularly important because mean surface solar irradiance is significantly greater (722 W m^−2^; *P* < 0.0001) in drylands compared to other biomes (512 W m^−2^) (Supplementary Information Fig. S1). Thus, changes in dryland albedo have the potential to disproportionately affect global energy balance as well as temperatures^26^,^27^.

As with other ecosystems, arid and semiarid ecosystem albedo is substantially influenced by natural and anthropogenic landscape change^28^. For example, land cover changes due to disturbance, such as deforestation^29^, wildfires^30^, conversion to croplands^31^, and climate change^32^, have been found to alter regional and even global climate by changing surface energy fluxes (i.e., albedo and radiative forcing)^28^,^33^. Increases in surface albedo due to anthropogenic historical (past 150 years) land use changes, particularly in North America, have been found to effect global effective radiative forcing and may have a larger impact on current global climate change than previously thought^33^. Increasing albedo linked to increasing global dryland cover, primarily as a result of desertification processes, has also been linked to a negative forcing at Earth’s surface, estimated to be equivalent to roughly 20% of the global anthropogenic CO_2_ effect over the past several decades^34^. However, understanding how shifts in biotic community cover—like those identified in our study—relate to overall biophysical feedbacks to climate requires consideration of how biotic community state changes impact not only albedo, but C sequestration from the atmosphere as well. For example, forest cover and albedo in semiarid drylands act as a positive forcing that outpaces the negative forcing linked to forest tree C sequestration^34^. In our study system, biocrust community shifts toward cyanobacterial dominance has been previously shown to reduce C uptake^6^,^7^, indicating potentially opposing effects on radiative forcings. Thus, the net effect of climate and land cover change will depend upon the relative magnitude of these different biophysical forcings and their interaction.

With this in mind, historical land use practices that directly impact dryland biocrusts (e.g., grazing, wildfire, land conversion) may have had past influence on the global climate, as well as produced subsequent feedbacks to climate change as in increased aerosol emissions and surface albedo^33^,^35^. In addition to direct effects of albedo on energy balance, changes in albedo can have large effects on global hydrological processes by accelerating snow melt and evapotranspiration from plants and soil^36^. Further, soil surface albedo can impact C cycling in precipitation limited, pulse-dynamic systems such as drylands via rapid effects on soil-water availability and surface soil temperatures, which are regulators of soil heterotrophic CO_2_ respiration and vegetation establishment and health^37^,^38^,^39^,^40^.

A rise in dryland surface albedo could create a large impact on the mean global albedo, where slight alterations greatly affect global climate patterns^41^, and this could be of growing concern with the degradation and disturbance of dryland biocrusts observed globally^6^,^9^,^10^,^42^,^43^,^44^,^45^,^46^. To explore the global effects of rising dryland surface albedo, we utilized the MODIS/Köppen ecosystem classification scheme from Elbert *et al*.^47^, which examined the distribution of biocrusts and their impacts on global N and C cycles^47^, to extract the global area of desert ecosystems containing biocrusts. We coupled this estimate with the data collected in our study to calculate an initial estimate of global mean radiative forcing (RF) produced from albedo changes resulting from a shift in biocrust community composition due to climate change.

**Figure 4:**
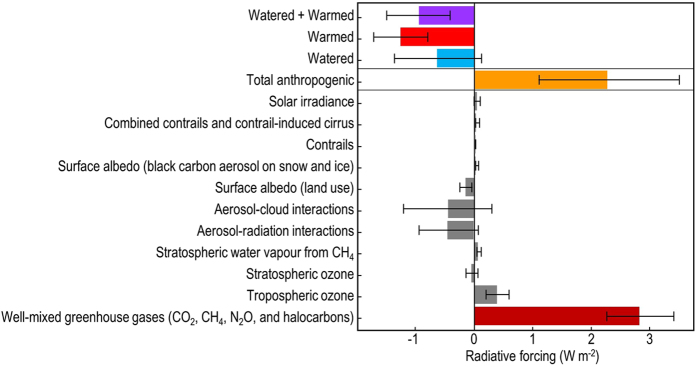
Estimated global mean radiative forcing from anticipated changes in biocrust communities and IPCC AR5 from 1750 to 2011. Global radiative forcing values resulting from changes in biocrust cover were calculated using equation (1) described in the methods. Uncertainties for treatment radiative forcing are represented by 95% confidence intervals (error bars). Effective radiative forcing (ERF) values were used for total anthropogenic, aerosol interactions, and well-mixed greenhouse gases. All other IPCC derived values are of radiative forcing (RF). Uncertainties for the IPCC AR5 RF and ERF values are represented by 5 to 95% confidence intervals. The blue, red, and purple bars show potential radiative forcing effects of climate-induced changes to biocrust communities in deserts. The maroon bar shows the positive radiative forcing resulting from increased greenhouse gas concentrations in the atmosphere, and the orange bar shows the effect of all anthropogenic sources combined (not including the albedo effects of biocrust community change).

Drylands encompass various MODIS/Köppen classifications beyond deserts, such as semiarid steppe ecosystems where biocrusts also inhabit shrub interspaces^48^. Here we used only the desert classification (with extremely arid regions, rock outcrops, and shifting sands eliminated from our calculations) and a desert biocrust cover correction factor (40%) to generate an estimate of change in global dryland radiative forcing due to a shift in biocrust community states [see Supplementary Information for detailed methods and Equation (1) for radiative forcing estimates]^47^. In Fig. 4, we offer a comparison of this calculated change in radiative forcing in the context of the IPCC AR5 (Intergovernmental Panel on Climate Change Fifth Assessment Report) global mean RF/ERF (effective radiative forcing) values^49^,^50^.

We stress that this comparison is not meant to be quantitative or predictive, but instead is provided to add insight into the potential for changes to biocrust cover to affect future climate via changes in radiative forcing on a global scale. The impact of climate change across all drylands may not be similar due to historical climatological and specific community differences. For example, cold deserts like the one studied here may be more or less sensitive to a changing climate than hot desert systems. To expand on this idea, we also calculated a change in radiative forcing for cold deserts, which comprise roughly 25.7% of the total desert global land surface area^51^. This calculation suggests that the warming of cold deserts alone could produce an estimated radiative forcing of −0.33 W/m^2^ derived from values found in Fig. 4. Much more work is needed to quantify these effects and the variability in their magnitude across systems, and we hope these results will help drive this line of questioning. Given this caveat, the estimated potential forcing from climate change as evaluated by our treatments is similar in size (but opposite in sign) to the forcings of greenhouse gas emissions and total anthropogenic effects (industrial and vehicle CO_2_ emission, land-use change, cropland irrigation, etc.). The net negative radiative forcing from climate change disturbances on biocrusts may indeed be acting to cool the surface, by returning radiation to the atmosphere that historically would have been absorbed at the Earth surface by the darker-colored, late-successional biocrust communities.

Strikingly, state transitions of biocrusts in response to climate change and physical disturbances, which lead to similar alternate community states^9^, have the potential to generate a large negative feedback to warming from climate change. However, we stress that this negative feedback comes at the expense of substantial losses of soil stability and altered biogeochemical functions that negatively impact soil fertility and reduce CO_2_ uptake from the atmosphere. Moreover, increased dust emissions following biocrust loss, and deposition to mountain and polar snow and ice, results in accelerated melting and reinforcement of the positive forcing from the snow-albedo feedback^52^,^53^. Dust itself, however, can heat or cool the atmosphere depending on composition and grain size characteristics^54^. Therefore, in addition to future efforts aimed at characterizing radiative forcing at larger scales across biologically-crusted systems, understanding to what extent biocrust degradation may already mask or contribute to rates of atmospheric warming through negative and positive feedback loops, respectively, remains a critical task.

## Methods

### Site Description and Field Study

The climate manipulation experiment located on the Colorado Plateau near Castle Valley, Utah, USA (38°38′4″N 109°24′38″W) contains twenty 5 m^2^ plots distributed across four treatments (control, watering, warming, and watering + warming) installed in 2005 in a randomized, complete block design (n = 5 for each treatment). Warming is achieved via infrared lamps that heated the topsoil to +2 °C above ambient at a soil depth of 1–2 cm from 2005–2008. To better match updated climate predictions, the temperature was increased to +4 °C above ambient in June 2008 to present. All plots received ambient precipitation, while watering and watering + warming treatment plots received additions of twice weekly 1.2 mm simulated monsoon (summer) rainfall events. An average of 35 simulated rainfall events occurred throughout the summer months from 2006–2012, equal to roughly four times the average natural frequency^8^. The warming and watering treatments reflect climate change model projections to 2050 for the study area^50^.

Plot albedos were estimated from directional reflectance measurements collected once from each plot from 29 October to 5 November 2014 using a spectro-goniometer system coupled with an Analytical Spectral Devices (ASD) FieldSpec 3 spectroradiometer. The spectro-goniometer system collected multiple spectral radiance (W m^−2^ nm^−1^ sr^−1^) measurements at specific zenith (−30°, −15°, Nadir/0°, 15°, 30°) and azimuth (0°, 45°, 270°, 315°) angles to first calculate hemispherical-conical reflectance factor (HCRF) spectra and then estimate albedo (Supplementary Information Fig. S5). The spectral radiance measurements covered the wavelength range of 350–2500 nm with 3–10 nm spectral resolution using an 8° field-of-view attachment.

Directional reflectance measurements were used to measure albedo rather than measuring albedo from hemispherical flux measurements to avoid contamination of albedo due to spatial heterogeneity that would affect the hemispherical field of view. Albedo estimates were made while surface soils were relatively dry and within ± 2 hours of solar noon. Albedo estimates were calculated from HDRF (hemispherical-directional reflectance factor) spectra by integration of the directional reflectance measurements (HCRF) across the hemisphere^55^.

Cyanobacteria, moss, and lichen cover was assessed in 40 cm × 40 cm gridded point-intercept frames (4 frames per plot surveying a total of 6400 cm^2^) during the autumn of 2014. Proportional cover for cyanobacteria within each plot was calculated as the ratio of points intercepting cyanobacteria to total biocrust cover. Proportional cover and statistical comparisons of these three groups among treatments can be found in Supplementary Information Fig. S6. Soil surface texture was measured prior to characterization of albedo (February 2014) using the chain method^56^ to determine the soil surface roughness upslope and across slope within each plot in order to generate a roughness index. This method^46^ fits a small-diameter, lightweight chain to the ground surface and compares the change in the linear distance of the fitted chain to the starting, fully-stretched chain length (i.e., a rougher surface results in a shorter distance, while a smoother surface results in a similar distance compared to the starting length). Plot soil moisture (volumetric water content or VWC) at a depth of 2 cm and 5 cm was measured with Campbell Scientific CS616 water content reflectometers and Decagon EC-5 volumetric water content probes. VWC values from both sensor types were reported as the hourly average of values recorded every five minutes ± 2 hours of solar noon on the same day as the assessment of plot albedo.

### Data Analysis

Albedo values among plots met the assumption of normality, but failed the ANOVA assumption of homoscedasticity among treatment groups. Thus, we used simple linear regressions to related albedo to individual surface properties and multiple linear regression to related albedo to combined ground surface properties (cyanobacterial, moss, and lichen cover; soil moisture at 2 and 5 cm, and surface roughness) in the absence of treatment groupings; and used a non-parametric Kruskal-Wallis test to compare albedo among treatment groups with Steel-Dwass post-hoc pairwise comparisons (a non-parametric test that corrects for sequential comparisons similar to Tukey’s HSD) for individual treatment contrasts. The best fit multiple regression model (i.e., the model that explained the most variation in albedo using the fewest independent factors) was determined as the model with the smallest Bayesian information criterion (*BIC*) value via a stepwise, forward fitting procedure. Albedo was also modeled with cyanobacteria, soil roughness, and soil moisture via a structural equation model. Albedo measured on one watering treatment plot was considered to be a high-end outlier, due to an error while collecting the HCRF measurements in the field and was subsequently removed before analyses to avoid biasing our estimates of climate-manipulation treatments on albedo. However, this outlier is retained in a companion set of analyses using identical testing procedures for readers’ consideration within Supplementary Information Figs S2–S4. All statistical procedures were completed in JMP Pro 12.0.1^57^, with the exception of the SEM, which was constructed with the GLS estimator and vetted via minimum function test statistic and *P*-values using the *lavaan* package in R version 3.1.2^58^,^59^.

## Additional Information

**How to cite this article:** Rutherford, W. A. *et al*. Albedo feedbacks to future climate via climate change impacts on dryland biocrusts. *Sci. Rep.* **7**, 44188; doi: 10.1038/srep44188 (2017).

**Publisher's note:** Springer Nature remains neutral with regard to jurisdictional claims in published maps and institutional affiliations.

## Supplementary Material

Supplementary Information
