# Supplementary material for: Albedo feedbacks to future climate via climate change impacts on dryland biocrusts
Source: Sci Rep. 2017 Mar 10;7:44188. doi: 10.1038/srep44188 (PMC5345002; doi:10.1038/srep44188)
Supplement: Supplementary Information [file srep44188-s1.pdf]

Supplementary Information

Albedo feedbacks to future climate via climate change impacts on dryland biocrusts

William A. Rutherford[1,2,*], Thomas H. Painter[3], Scott Ferrenberg[1], Jayne Belnap[1], Gregory S. Okin[4], Cody Flagg[5], Sasha C. Reed[1]

[1]United States Geological Survey, Southwest Biological Science Center, Moab, UT 84532 USA
[2]Current address: School of Natural Resources and the Environment, University of Arizona, Tucson, AZ 85721, USA
[3]Joint Institute for Regional Earth System Science and Engineering, University of California, Los Angeles, CA 90095 USA
[4]Department of Geography, University of California, Los Angeles, CA 90095 USA
[5]National Ecological Observatory Network (NEON), Boulder, Colorado 80301 USA

[*]Corresponding author address: School of Natural Resources and the Environment, University of Arizona, Tucson, AZ 85721, USA; arutherford@email.arizona.edu

Table S1. Percent change from control for soil surface roughness and moisture. Percent change was calculated from the treatment averages for both soil roughness and moisture. For soil roughness, a negative value indicates a smoother soil surface from the control. Negative soil moisture indicates a dryer soil surface compared to the control. The soil roughness percentage values are derived from a soil roughness index calculated following Saleh 1993. The soil moisture percentages were calculated from direct measurements of volumetric water content (see Methods).

Treatment	Soil Roughness (%)	Soil Moisture (%)
Warmed	-54.23	-0.78
Watered + Warmed	-55.26	-2.86
Watered	0	7.9

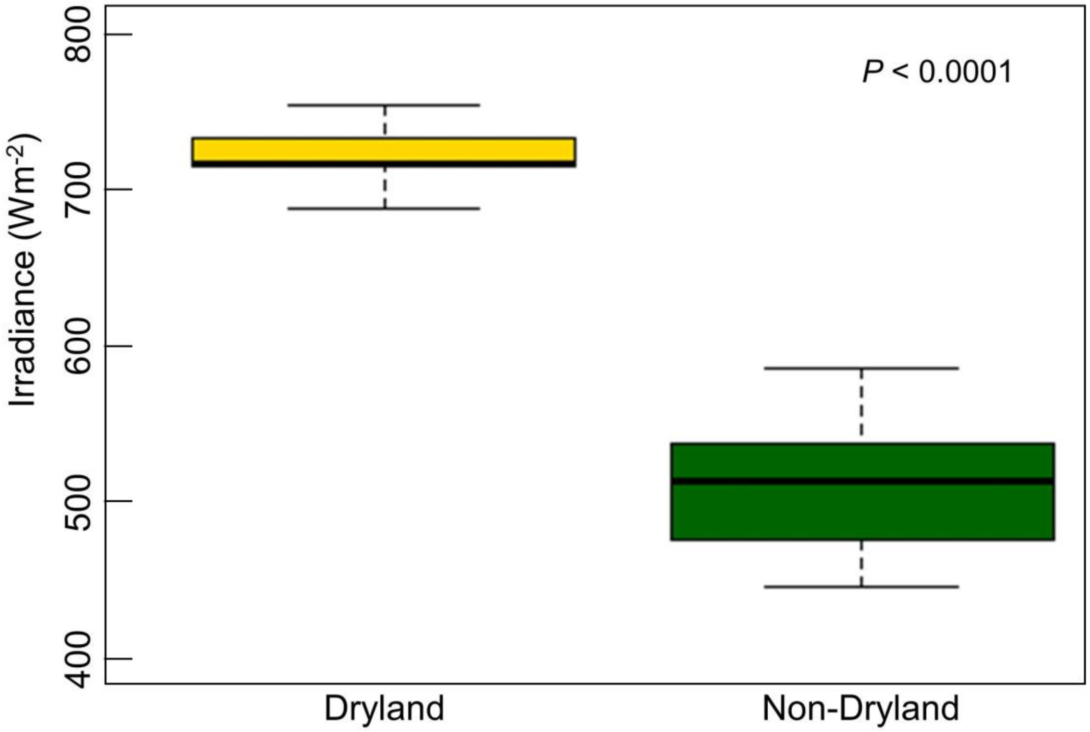

Figure S1: Boxplot of at surface irradiance in drylands and non-drylands in the USA Global Horizonal Irradiance (W m^{-2}) data compiled from 10 different Department of Energy NREL (National Renewable Energy Laboratory) locations (*n*=5 for dylands; *n*=5 for non-drylands) in 2012. The monthly average irradiance in 2012 was averaged for a yearly average irradiance value. Drylands significantly differed in surface irradiance from non-dryland locations ($P < 0.0001$). Significant differences between dryland and non-dryland locations were tested with a one-way ANOVA using the statistical program R.

Calculation used in estimation of radiative forcing values

Using the experimental albedo values ($\alpha_{control}$, $\alpha_{treatment}$), a modified equation was used to estimate radiative forcing (RF):

$$RF = E_{sun} * (\alpha_{control} - \alpha_{treatment}) * (A_{desert}/A_{earth}) * (0.4) \quad (1)$$

where E_{sun} (W/m^2) is the integrated ASTM (American Society for Testing and Materials) G-173-03 direct plus circumsolar earth surface solar energy derived from the NREL SMARTS v 2.9.2 (National Renewable Energy Laboratory Simple Model of the Atmospheric Radiative Transfer of Sunshine) standard[59]. Radiative forcing values were corrected for global drylands (A_{desert}/A_{earth}) using the desert ecosystem MODIS/ Köppen[60] classification area (A_{desert}) ($13.75 x 10^{12}$ m^2) that excluded areas with annual precipitation <75 mm and areas designated as “dune sand/shifting sands” and “rock outcrops” [47] where biocrusts are not likely to form, divided by the total surface area of the earth (A_{earth}) ($5.101 x 10^{14}$ m^2)[61]. The resulting RF value was corrected for approximate ground biocrust cover (40% in desert biome) [47]. Confidence intervals (95%) were calculated for our globally corrected radiative forcing values. Treatment estimated radiative forcing values were plotted against 1750-2011 global mean RF and effective radiative forcing (ERF) values reported in the 2013 IPCC AR5 (Intergovernmental Panel on Climate Change Fifth Assessment Report) [49,50] (Fig. 3, S4).

The following references are specific only to the Supplementary Information radiative forcing calculation above and not also cited in the manuscript:

60. NREL, A. M. 1.5 Gobal solar spectrum derived from SMARTS v. 2.9. 2.
61. Friedl, M. A. *et al.* Global land cover mapping from MODIS: algorithms and early results. *Remote Sens. Environ.* **83,** 287–302 (2002).
62. Trenberth, K. E. *Climate system modeling*. (Cambridge University Press, 1992).

The following three figures (S2-S4) include the outlier in the dataset. These figures display the large variance in the watering treatment (Figure S2 and S4) and can be used to compare with the figures in the manuscript, which do not include the outlier. Figure S3 has the same R^2 and *P*-value independent of outlier removal.

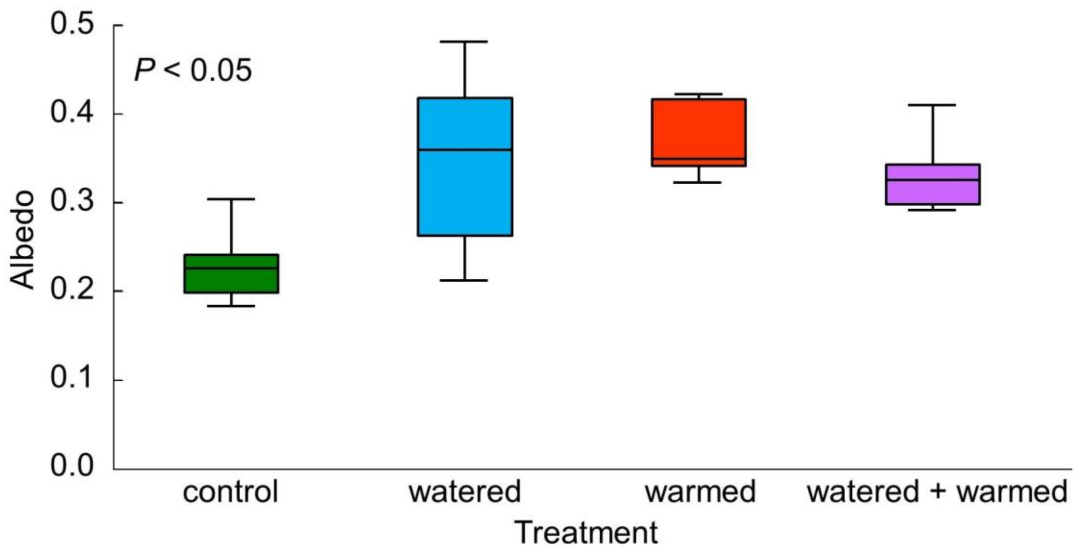

Figure S2 | Boxplot of albedo separated by treatment with outlier in Water treatment
Significant differences were found between the control albedo and the warm ($P < 0.01$) and water + warm ($P < 0.05$) treatments, but did not significantly differ from the water treatment ($P = 0.09$) with the outlier included. The large variance, due to possible measurement error, in the water treatment led to the conclusion to remove the outlier and use the Kruskal-Wallis analysis of variance.

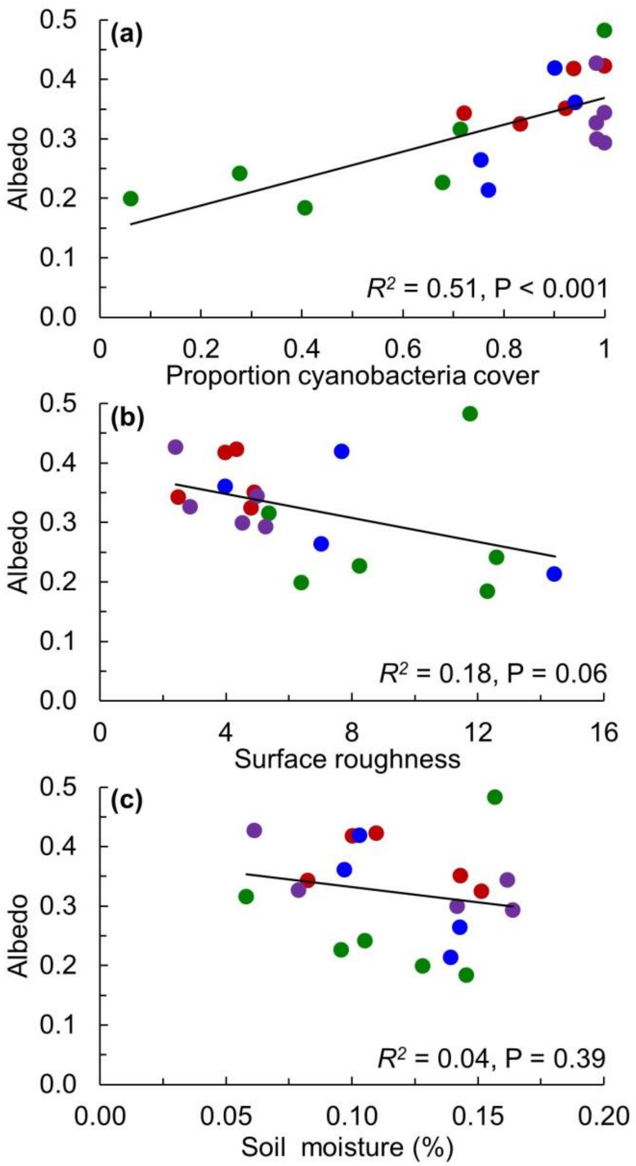

Figure S3 | Linear models relating albedo to the proportional cover of cyanobacteria, soil surface roughness and soil moisture

The (a) proportional cover of cyanobacteria within biocrust communities of experimental plots was collected in point-intercept frames in autumn 2014, and calculated as the ratio of points intercepting cyanobacteria relative to total biotic cover (sum total of cyanobacteria, moss, and lichen points). Soil surface roughness (b) was measured in spring 2014 to calculate a roughness index to characterize the soil surface roughness upslope and across slope within each plot. Soil moisture (c) at a depth of 5 cm was measured as the hourly average of values recorded every five minutes during the same time as the albedo measurements. R^2 and *P*-values are from simple linear regression. Climate treatments are denoted by symbol colors (green = control, red = warming, blue = watering, purple = warming + watering). The outlier in the water treatment is included in this analysis (n = 5 compared to Figure 2 where n = 4).

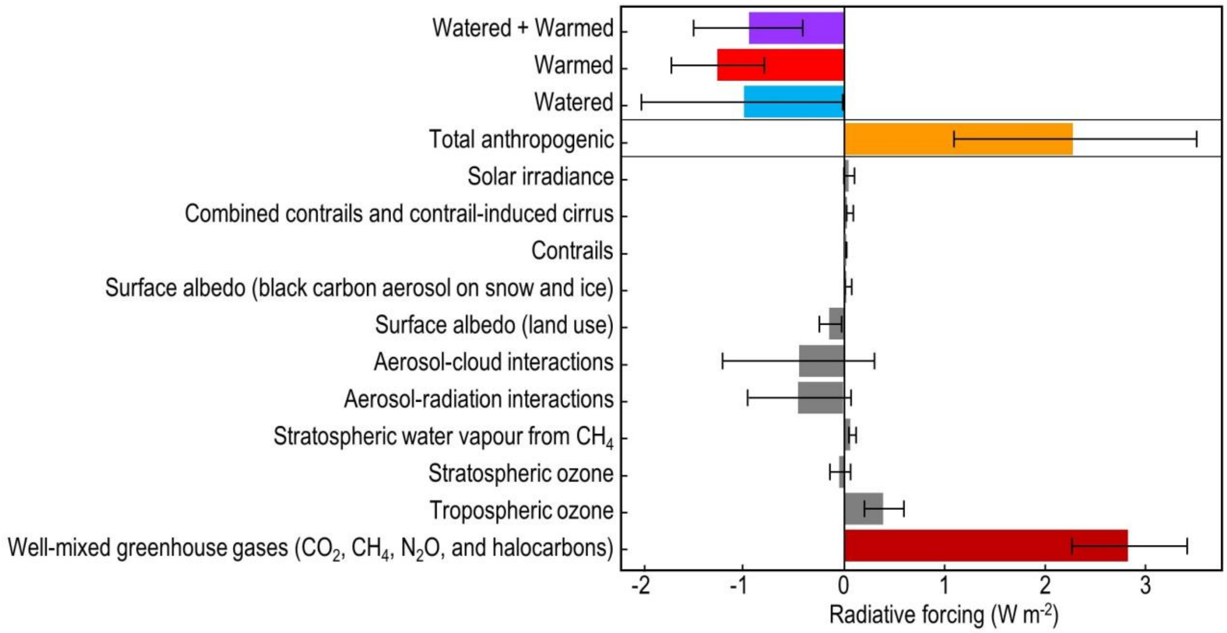

Figure S4 | Estimated global mean radiative forcing from anticipated changes in biocrust communities and IPCC AR5 from 1750 to 2011. Global radiative forcing values resulting from changes in biocrust cover were calculated here using all data (i.e., the data shown in Figure 3 plus the noted outlier for the watered treatment) in equation (1) described in the methods. Uncertainties for treatment radiative forcing are represented by 95% confidence intervals (black bars). Effective radiative forcing (ERF) values were used for total anthropogenic, aerosol interactions, and well-mixed greenhouse gasses. All other IPCC derived values are of radiative forcing (RF). Uncertainties for the IPCC AR5 RF and ERF values are represented by 5 to 95% confidence intervals.

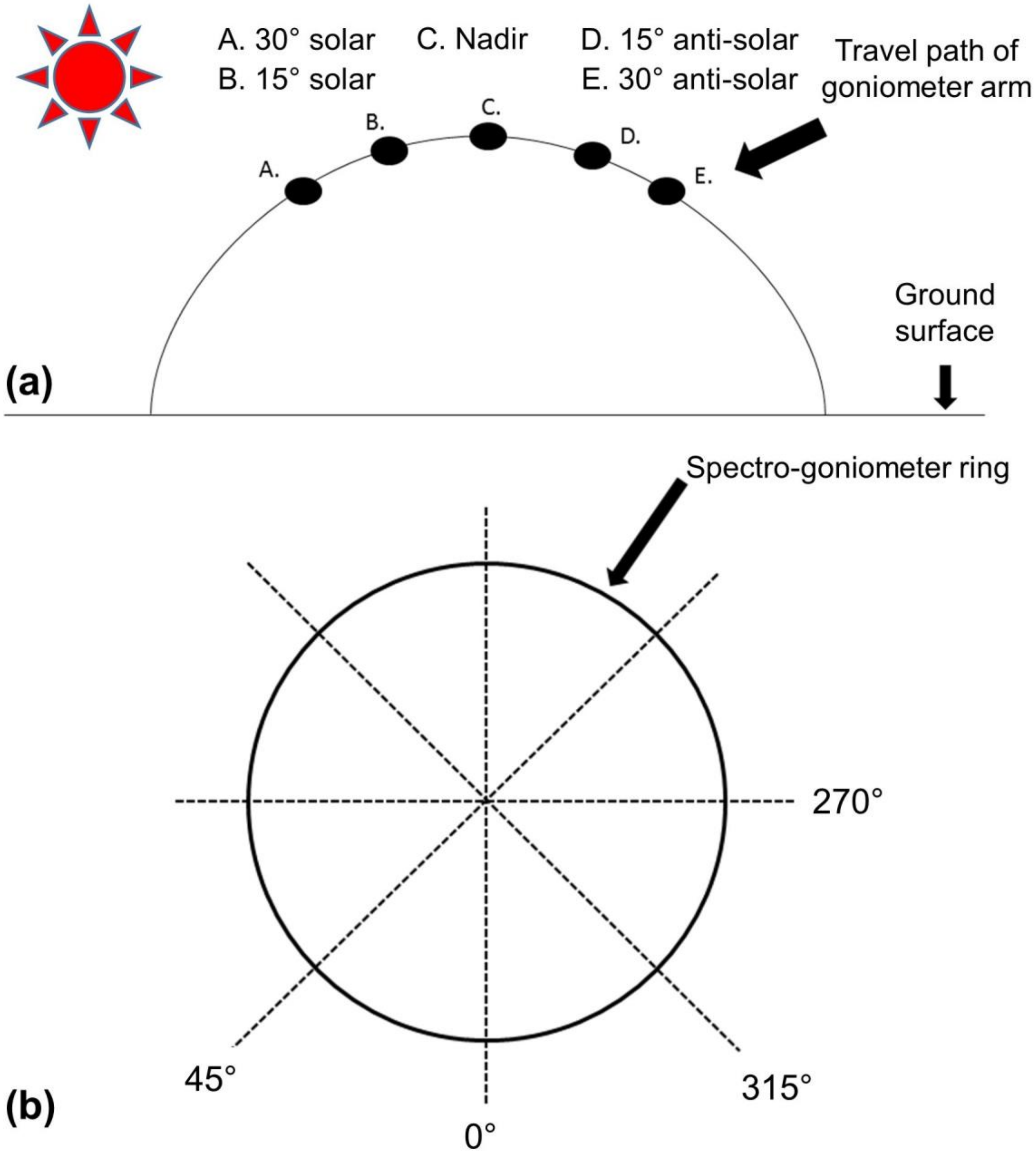

Figure S5 | Diagram of spectro-goniometer collection angles (zenith and azimuth)
Spectra were collected at several, repeated zenith (**a**) and azimuth (**b**) angles to more accurately estimate albedo. Central zenith angles along the spectro-goniometer arc were chosen to limit the amount of interference from plot instrumentation and non-soil surfaces (e.g., vascular plants). Specific azimuth angles (or planes) were used to capture the maximum amount of scattering reflected light.

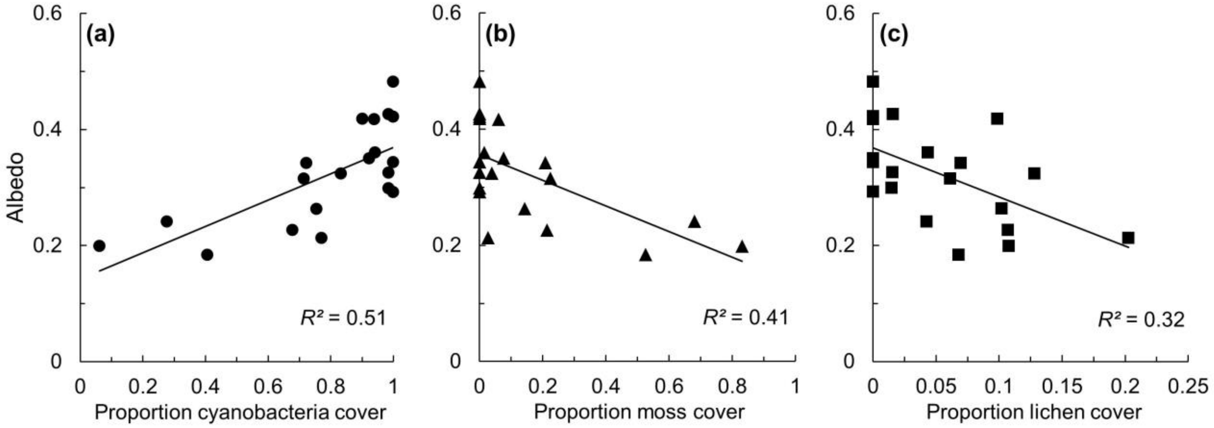

Figure S6: Linear regression models relating albedo to surface cover of key biocrust organism groups

The proportional cover of dominant biocrust community groups was collected via point-intercept frames in autumn 2014, and calculates the ratio of points intercepting cyanobacteria (a), moss (b), or lichen (c) to total biotic cover (sum total of cyanobacteria, moss, and lichen points). Albedo was significantly related to all three biocrust community groups, with cyanobacterial ground-surface cover explaining the largest amount of variation in albedo.
